# Antiseptic Pocket-Case

**Published:** 1888-04

**Authors:** 


ANTISEPTIC POCKET CASE.—The necessary elements in antisepsis are soap, a brush, and an antiseptic, the latter capable of accurate dosage, and colored, that it may not be mistaken for something else. One finds soap in every house, but the other two elements must be carried. Dr. Egli-Sinclair recommends, and has used satisfactorily for months, a case containing a nail-brush and a bottle containing the following solution:

| | |
|---|---|
| Hydrarg. chlor. corros | 40.0 |
| Ammon. chlorat | 8.0 |
| Nacht blau | 0.8 |
| Aquæ distillatæ | 200.0 |

The stopper of the bottle is hollow, and holds enough of the fluid to make, when added to one litre (about two pints) of water, a 0.1 per cent. sublimate solution, strongly colored blue, which even a layman can not fail to recognize. Such a case is especially commended to obstetricians and midwives.—*Correspondenz—Blatt für Schweizer Aerzte.*

